# Neutropenic Enterocolitis Complicating Induction Chemotherapy in an Acute Myeloid Leukemia Patient

**DOI:** 10.7759/cureus.13029

**Published:** 2021-01-31

**Authors:** Kevin Groudan, Audrey Ready, Riffat Sabir

**Affiliations:** 1 Internal Medicine, Baystate Medical Center, Springfield, USA

**Keywords:** neutropenic enterocolitis, typhilitis, chemotherapy, acute myeloid leukemia

## Abstract

Neutropenic enterocolitis is a rare inflammatory condition of the ileocecum. Clinicians should be aware of neutropenic enterocolitis in neutropenic patients with hematologic malignancies undergoing chemotherapy as it portends a poor prognosis if not diagnosed early in its course. We report a patient diagnosed with neutropenic enterocolitis within one week of receiving induction chemotherapy for acute myeloid leukemia.

## Introduction

Neutropenic enterocolitis is a rare inflammatory condition of the ileocecum. Clinicians should be aware of neutropenic enterocolitis in neutropenic patients with hematologic malignancies undergoing chemotherapy as it portends a poor prognosis if not diagnosed early in its course. We report a patient diagnosed with neutropenic enterocolitis within one week of receiving induction chemotherapy for acute myeloid leukemia. 

## Case presentation

A 53-year-old man with history of hypertension, hyperlipidemia, diabetes mellitus, nephrolithiasis, and hypothyroidism presented to the ED with several months of generalized weakness, unintentional weight loss, and exertional dyspnea. His vitals were significant only for tachycardia and exam revealed a 3 cm firm posterior cervical lymph node and splenomegaly. Laboratory work was notable for a white blood cell count of 191.5 k/mm3 with 39% blasts, hemoglobin of 4.3 g/dL, platelet count of 24 k/mm3, INR of 1.3, and a creatinine of 2 mg/dL from a baseline of 1.0 mg/dL. A peripheral smear showed multiple blasts with heterogenous scant cytoplasm concerning for acute myelogenous leukemia. A bone marrow biopsy showed hypercellular marrow with blasts comprising 55% of the aspirate differential, confirming this diagnosis. The patient was started on induction chemotherapy with daunorubicin (106.2 mg every 24 hours for three doses) and cytarabine (177 mg every 24 hours for seven doses).

**Figure 1:**
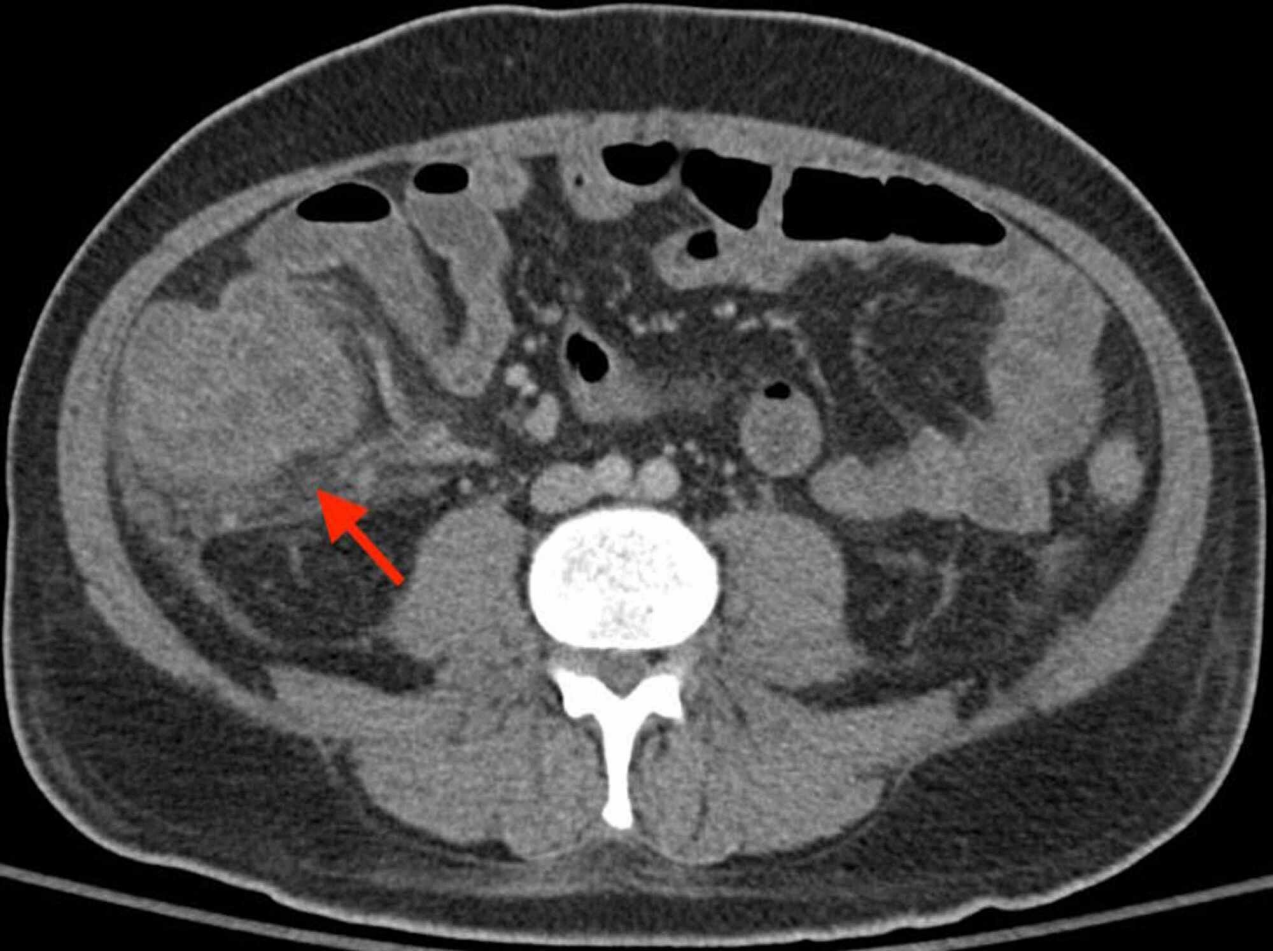
CT showing marked wall thickening involving the cecum and right hemicolon, consistent with neutropenic enterocolitis.

The patient's leukocytosis down-trended significantly. By day 5, he was neutropenic, with a white blood cell count of 0.4 k/mm3 and absolute neutrophil count of 0.1 k/mm3. On day 6, he developed a low-grade fever of 100.7°F and had diarrhea. Cefepime was started for neutropenic fever. However, the patient remained febrile and developed profuse diarrhea. Blood cultures subsequently grew *Escherichia coli*, concerning for a gastrointestinal or genitourinary etiology. A CT abdomen and pelvis (CT a/p) with IV contrast ordered revealed marked wall thickening involving the cecum and right hemicolon, consistent with neutropenic enterocolitis (Figure 1).

The patient was switched to IV meropenem and completed a 27-day course with normalization of his blood cultures and resolution of his fever and diarrhea.

## Discussion

Neutropenic enterocolitis, also known as typhilitis, from the Greek word “typhlon” or cecum,” refers to a severe inflammatory condition of the ileocecum [1]. It primarily occurs in neutropenic patients with hematologic malignancies; however, it has also been reported in immunocompromised patients with AIDS and organ transplant [2]. Its incidence is estimated to be 5.6% in hospitalized adults with hematologic malignancies [3]. Patients commonly present with fever, abdominal pain, nausea, vomiting, or diarrhea in the setting of a neutrophil count less than 0.5 k/mm3 in the third week of chemotherapy [4-5]. Our patient developed symptoms of fever and diarrhea in the first week of chemotherapy and had a neutrophil count of 0.1 k/mm3.

The pathogenesis of neutropenic enterocolitis is poorly understood. Gut mucosal injury from cytotoxic chemotherapy, in combination with profound neutropenia, is thought to lead to impaired host defenses and microorganism invasion [6]. Predilection for the cecum is thought to be due to its reduced vascularization relative to the rest of the colon [7]. Common culprit microorganisms include Gram-negative bacilli, Gram-positive cocci, anaerobes, and Candida species [7]. Bacterial translocation and bacteremia is a common sequelae of neutropenic enterocolitis [7], as was seen in our patient.

Neutropenic enterocolitis is diagnosed by CT of the abdomen, which reveals cecal wall thickening, mucosal enhancement, bowel dilation, or pneumatosis [8]. CT helps delineate from commonly presenting diagnoses such as pseudomembranous colitis, ischemic colitis, appendicitis, inflammatory bowel disease and other infectious causes [2]. Blood and stool cultures should also be obtained to guide antibiotic management, and *Clostridium difficile* toxin should be ruled out as cytotoxic disruption of the gut predisposes to its infection [2].

Patients are managed with antimicrobial therapy and bowel rest [2]. Although no large studies have been published on the prognosis of neutropenic enterocolitis, early reports had quoted the mortality as 50% or greater [9]. With development of imaging modalities and early recognition, mortality has reduced substantially. In fact, one case control study performed between 1995 and 2005 studied 42 children with neutropenic colitis and all 42 recovered with treatment [10].

## Conclusions

In conclusion, we present a rare case of neutropenic enterocolitis in a patient with newly diagnosed acute myeloid leukemia undergoing induction chemotherapy. Neutropenic enterocolitis is a life-threatening infection which providers should always consider in patients with fever, abdominal distention, or diarrhea undergoing chemotherapy.
